# Clinical characterization of patients and genomic description of respiratory syncytial virus in adults and paediatric patients in a northeastern Mexican population

**DOI:** 10.1099/jmm.0.002154

**Published:** 2026-06-05

**Keywords:** lower respiratory tract, Mexico, pneumonia, respiratory syncytial virus

## Abstract

**Introduction.** Respiratory syncytial virus (RSV) is a frequent cause of lower respiratory tract infections among children under 2 years old and adults over 60. Compared to other causes of viral pneumonia in adults, it is associated with a higher risk of intensive care unit admission, mechanical ventilation and 30-day mortality.

**Hypothesis/Gap Statement.** Despite the global recognition of RSV as a cause of respiratory disease, the epidemiological information available on its distribution in Mexico and Latin America among adults is limited.

**Aim.** To characterize the epidemiological, clinical and molecular features of RSV infections in the Mexican population during the 2023–2024 winter season.

**Methodology.** An observational, retrospective and descriptive study was conducted including patients of all ages with RSV detected by reverse transcriptase/real-time PCR during the 2023–2024 winter season.

**Results.** In our study, 50% of adults and 96% of paediatric patients with RSV were admitted for at least 24 h. The most frequent comorbidities in adults were systemic hypertension and diabetes. Approximately one-third of adults were over 65 years of age, and the overall in-hospital mortality in the adult group was 24%, in contrast to 6% in-hospital mortality in the paediatric population. Genomic analysis identified RSV serotype A as predominant. Complete genome sequencing revealed circulation of the A.D.1 clade, consistent with lineages from the southern USA, marking the first complete sequencing of RSV isolates from Mexican patients.

**Conclusion.** RSV represents a significant cause of respiratory infection in Mexico across age groups, with substantial mortality in adults. The identification of the A.D.1 clade underscores the importance of continuous epidemiological and molecular surveillance for understanding RSV circulation patterns in the region.

**Impact Statement**

Respiratory syncytial virus (RSV) is a leading cause of serious respiratory illnesses worldwide, especially among children under 2 years old and older adults. Although its epidemiology has been well studied in high-income countries, there is a significant gap in comprehensive data from Latin America, particularly for adults. This study offers the first detailed epidemiological overview of RSV infections across all ages in Mexico during the 2023–2024 winter season, incorporating clinical, demographic and genomic data. We highlight a high rate of hospitalized RSV cases, common comorbidities and notable differences in mortality between children and adults. Additionally, we provide the first full genome sequence of RSV from Mexican patients, identifying it as part of the A.D.1 clade, closely related to strains from the southern USA. These insights broaden the global understanding of RSV diversity and its circulation, guiding public health strategies locally and regionally and supporting genomic surveillance in the Americas. Our findings emphasize the urgent need for ongoing RSV monitoring in Latin America to enhance clinical outcomes and prepare for new preventive options available such as vaccines and monoclonal antibodies in national health programmes.

## Data Summary

The complete genome sequences of the Human respiratory syncytial virus A isolates used in this study have been deposited in GenBank under accession numbers PP790963.1 (RSV-A/MX/UANL-92777/2023, https://www.ncbi.nlm.nih.gov/nuccore/PP790963.1), PQ599899.1 (isolate 93484, https://www.ncbi.nlm.nih.gov/nuccore/PQ599899.1) and PQ599900.1 (isolate 93557, https://www.ncbi.nlm.nih.gov/nuccore/PQ599900.1). No custom code or software was generated for this study.

## Introduction

Respiratory syncytial virus (RSV) is the main pathogen responsible for lower respiratory tract infections among children under 2 years of age [1]. In adults over 60, the incidence of community-acquired RSV infections ranges from 3% to 7% in high-income countries [2]. Various studies have described a clear association between complications and the risk of hospitalization in adults with comorbidities (chronic obstructive pulmonary disease, cardiovascular diseases, chronic kidney disease, diabetes, etc.) and individuals over 75 years of age [3,4]. Compared to other causes of viral pneumonia in adults, RSV infection has been associated with a higher risk of admission to the intensive care unit (ICU), mechanical ventilation and 30-day mortality [5].

RSV is an RNA virus that belongs to the *Paramyxovirus* family. Based on the G glycoprotein, sequencing can be classified into two subtypes, A and B [6]. Worldwide, 13 distinct genotypes have been identified for subtype A, while subtype B has at least 37 recognized genotypes. The most prevalent genotypes are NA1 (76.30%) for subtype A and BA (70.65%) for subtype B [7]. Both genotypes circulate and fluctuate during the winter seasons, and ongoing monitoring and sequencing allow for a comprehensive understanding of their diversity [8].

Despite the global recognition of RSV as a significant cause of respiratory disease, there is limited epidemiological information regarding its distribution in Latin America, particularly among adults [9]. A 2016 study estimated that the incidence of RSV infection was 4.6% in adults and 4.7% in children in Mexico. Notably, the infection was more prevalent in children under 5 years of age, with 66% of the reported cases in that age group [10]. Also in Mexico, samples from paediatric patients with RSV infection were sequenced, showing the predominance of the RSV-A subtype and the presence of the ON1 genotype [11].

Molecular surveillance approaches are fundamental for improving comprehension of the incidence and diversity of RSV. Currently, there is limited information describing how the genetic diversity of the virus influences clinical outcomes in adult patients.

In this study, we described the genomic and clinical characteristics of adult and paediatric patients from northeastern Mexico during a single winter season.

## Methods

### Study site

The Infectious Diseases and Hospital Epidemiology teams evaluated and tested patients at the Dr. José Eleuterio González University Hospital of the Universidad Autónoma de Nuevo León. The virus was genetically analysed at the Medical Virology Research and Innovation Center (CIIViM).

### Study population

Patients of all ages presenting with respiratory symptoms suspected to be of viral aetiology were evaluated. Nasopharyngeal swabs were collected for reverse transcription quantitative polymerase chain reaction(RT-qPCR) testing using the Allplex™ [Severe Acute Respiratory Syndrome Coronavirus 2 (SARS-CoV-2)/FluA/FluB/RSV Assay] to detect SARS-CoV-2, Influenza and/or RSV infections. The emergency and epidemiology department assessed and interviewed patients before sample collection to determine the need for testing. A nasopharyngeal swab was considered if the patient presented with at least 1 day of two or more of the following symptoms: cough, rhinorrhoea, fever, headache, dyspnoea, myalgia, arthralgia or sore throat.

### Data collection

Data was collected using a standard case study format, which included demographic information, symptoms, comorbidities, influenza and SARS-CoV-2 vaccination history, treatment use prior to assessment and vital signs. Additional information was also collected in case of complications associated with viral infection, including treatment details, antibiotic use, need for respiratory support, ICU admission and in-hospital mortality.

### Sample processing

**Fig. 1.**
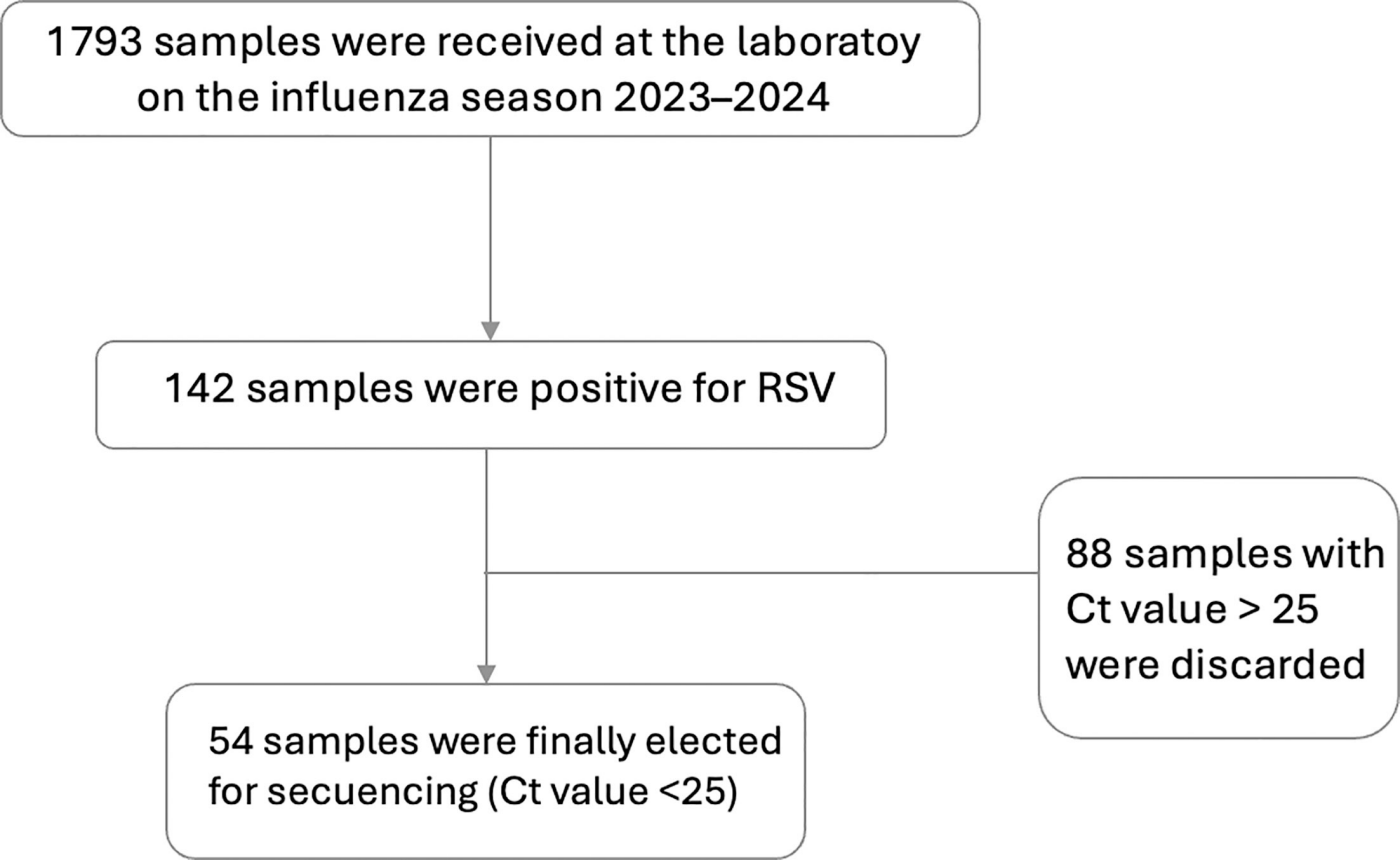
Flow chart of sample selection for sequencing.

Samples with an RSV detection result were stored at −80°C. Subsequently, samples with a cycle threshold below 24 were submitted for sequencing (Fig. 1).

We used two approaches to sequence the whole genome of RSV, sequence-independent single-primer amplification (SISPA) and sequence-specific amplification. The SISPA amplification was done as reported elsewhere [12]. The specific primer amplification was made using the primers reported by Langedijk that divide the whole genome into four fragments [13].

The sequencing library was prepared using the Rapid sequencing DNA v.14 barcoding (SQK-RBK114.24) (Oxford Nanopore Technologies, UK) and loaded into a Flongle Flow Cell (FLO-FLG114) on a MinION device (Oxford Nanopore Technologies). The basecalling and demultiplexing were done with Dorado v.0.6 (Oxford Nanopore Technologies) and the reads were aligned with an RSV-A reference genome (NC_038235.1), using minimap2 v.2.28 [14]. The consensus genomes were obtained with medaka v.1.8.1 (Oxford Nanopore Technologies) and bcftools v.1.17 [15]. The obtained sequences were deposited in GenBank (PP790963, PQ599899 and PQ599900). We used Nextstrain’s build for RSV for our analysis [16]. Briefly, Nextstrain is composed of two core parts: Augur (v.31.2.0) and Auspice (v.2.63.0). Augur contains the bioinformatic tools for analysis and Auspice is the web-based visualization program. A build is a program of several commands and data that produces a single dataset. The build made by Augur is in charge of filtering, aligning, inferring the phylogenetic tree, refining the tree and exporting the dataset. For aligning, it uses MAFFT v.7-526 [17]; phylogenetic tree inference IQ-TREE2 v.2.4.0 [18] and for time-scaling, dating and ancestral reconstruction; and TreeTime v.0.11.4 [19]. For the analysis, we used the curated metadata and sequences for RSV-A available in https://data.nextstrain.org and the whole-genome sequences from Mexico deposited in GenBank.

### Statistical analysis

Independent descriptive statistical analysis was performed for confirmed cases of RSV-A and RSV-B infection, including means for continuous variables and distribution for categorical variables. The chi-square test or Fisher’s exact test was used to compare the proportions of demographic and clinical characteristics. A *P*-value <0.05 was considered significant for all tests.

## Results

**Fig. 2.**
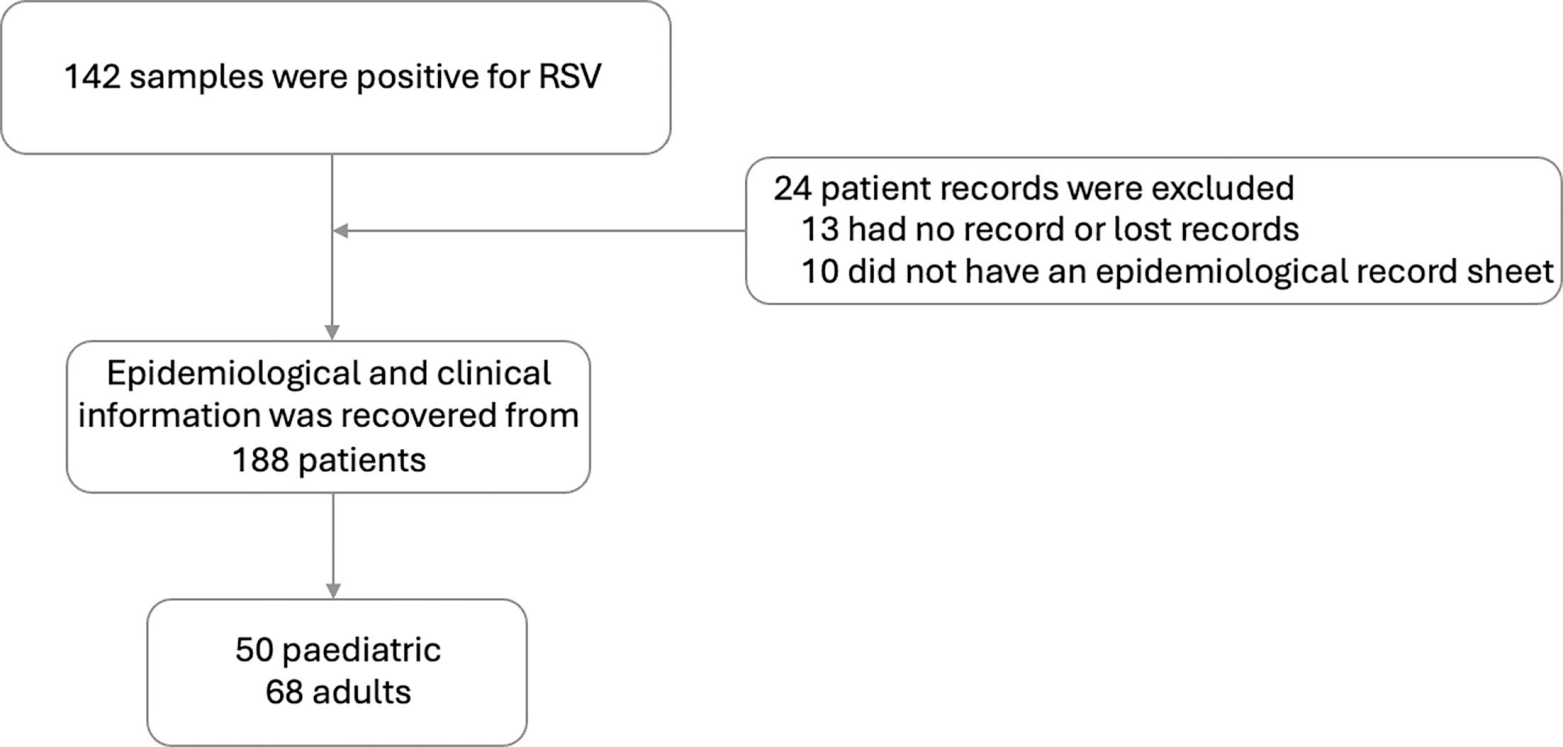
Flow chart of patients with positive RSV samples for clinical and demographic compilation.

From October 2023 to February 2024, 1,700 patients with respiratory symptoms were evaluated, of which 142 (8.3%) samples were detected for RSV by RT-qPCR. Clinical information was collected from 118 patients, as complete epidemiological records were unavailable for the remaining patients (Fig. 2). Of these patients, 57% (*n*=68) were 18 years old or older, and the remaining 42% (*n*=50) were paediatric patients.

Of the total, 50.08% (*n*=60) were adult males. In total, 27.12% (*n*=32) were healthcare workers; 60% (*n*=41) of adults had at least one comorbidity, with the four most common being systemic arterial hypertension 26.4% (*n*=18), type 2 diabetes and cardiovascular disease 16.18% (*n*=11) each and history of any type of immunosuppression (e.g., cancer patients and steroid use) 14.71% (*n*=10). Hospitalization was required for 50% (*n*=34) of the adults and 96% of the paediatric patients (at least for 24 h). Among outpatients, 96.9% (*n*=32) of adults were healthcare workers, and 51% (*n*=17) reported a history of influenza vaccination (Tables1  2).

**Table 1.** Demographic characteristics of patients

	*n*=118	Percentage (%)
Age, years, median		
Paediatric	50	1.2 (0–14)
Adult	68	38.93 (17-93)
Gender		
Male	60	50.8
Female	58	49.2
Healthcare workers	32	27.12
Symptoms and signs duration (days)	4.06 (±3.4 d)	
Cough	109	92.37
Rhinorrhoea	90	76.27
Dyspnoea	58	49.15
Fever	58	49.15
Expectoration	53	44.92
Headache	36	30.51
Odynophagia	33	27.97
Myalgias	33	27.97
Intercostal retraction	23	19.49
Vomiting	13	11.02
Diarrhoea	11	9.32
Conjunctivitis	9	7.63
Cyanosis	4	3.39

**Table 2.** Description of comorbidities in adult and paediatric populations

	Adult (*n*=68)	Paediatric (*n*=50)	*P*-value
With comorbidities (at least one)	60.29% (41)	22.00% (11)	**<0.001**
Hypertension	26.47% (18)	2.00% (1)	**<0.001**
Diabetes	16.18% (11)	0% (0)	**<0.003**
Cardiovascular disease	16.18% (11)	6.00% (3)	0.148
Immunosuppression*	14.71% (10)	6.00% (3)	0.233
Asthma	5.88% (4)	2.00% (1)	0.236
Tuberculosis	2.94% (2)	2.00% (1)	0.748
Hypothyroidism	2.94% (2)	0% (0)	0.221

* Patients with immunosuppression: cancer diagnosis, Human Immunodeficiency Virus (HIV) infection, steroid use and haematological malignancies. Fisher’s exact test. Bold case indicates significance.

Overall, the mean duration of symptoms was 4.06 (±3.4) days, and the most common were cough (92.37%), rhinorrhoea (76.27%), dyspnoea (49.15%) and fever (49.15%).

In the group of hospitalized adult patients, 57.5% (*n*=19) were women, with 41.4% (*n*=14) having a history of influenza vaccination and 33.3% (*n*=11) being over 65 years old. Oxygen supplementation was required in 30.3% (*n*=10), 15.1% (*n*=5) required non-invasive mechanical ventilation (NIMV), and 15.1% (*n*=5) needed invasive mechanical ventilation (IMV). ICU admission was reported in 6.0% [2]. Acute kidney injury was reported in 27.2% (*n*=9) of the patients, and the mean hospital stay was 7.4 days [interquartile range (IQR) 13] and in-hospital mortality was 24.2% (*n*=8) (Table 3).

**Table 3.** Comparison of clinical and epidemiological characteristics between adult and paediatric populations with RSV infection

Variable	Adult (*n*=68)	Paediatric (*n*=50)	***P*-value**
**Care setting**			
Outpatient treatment	50% (34)	4% (2)	<0.001
**Gender**			
Male	47.05% (32)	56.00% (28)	0.357
**Influenza vaccination**	45.58% (31)	0% (0)	<0.001
**Pulse oximetry at admission**			
≥95%	50% (34)	68.00% (34)	0.022
<90%	32.35% (22)	18.00% (9)	0.934
≤85%	8.82% (6)	10.00% (5)	0.999
**Coinfections**			
Influenza	1.47% (1)	4.00% (2)	0.573
SARS-CoV-2	0% (0)	2.00% (1)	0.423
Bacterial growth in culture	0% (0)	12.00% (6)	**0.004**
**Inpatient treatment**			
	*N*=34	*N*=48	**<0.001**
Oxygen requirement	30.3% (10)	52.08% (25)	**<0.001**
NIMV	15.1% (5)	29.16% (14)	**<0.001**
IMV	15.1% (5)	22.91% (11)	**0.029**
ICU admission	6.0% (2)	37.5% (18)	**<0.001**
Acute kidney injury	27.2% (9)	10.41% (5)	0.774
Length of hospital stay (median, IQR)	7.4 days (IQR 13)	8 days (IQR 11)	**0.004**
Mortality	24% (8)	6.00% (3)	0.351

Fisher’s exact test. Bold case indicates significance.

Paediatric patients in our cohort required at least 1 day of hospitalization (emergency, general ward and ICU) in 96% (*n*=48) of the cases, while only 4% (*n*=2) were treated as outpatients entirely. Among hospitalized patients, 56.3% (*n*=27) were male. In total, 87% (*n*=42) were less than 2 years of age. Of the paediatric patients, 80% (*n*=40) had no underlying medical conditions. A total of 6% (*n*=3) were children born to mothers with gestational diabetes, 6% (*n*=3) had some form of immunosuppression, 4% (*n*=2) had a diagnosis of bronchopulmonary dysplasia and two patients were reported as premature. In addition, 68% (*n*=34) had an oxygen saturation >95%, while 18% (*n*=9) had a saturation below 90%, and 10% (*n*=5) had <85% upon admission (Table 3).

Regarding hospitalized paediatric patients, two were co-infected with influenza A and one with SARS-CoV-2. These two patients were considered co-infected because they tested positive for both RSV and influenza A (*n*=1) and SARS-CoV-2 (*n*=1) by reverse transcriptase/real-time PCR and were classified as such due to the absence of documented prior respiratory symptoms in their medical records that could account for their hospitalization. Additionally, 25% (*n*=6) had bacterial growth in respiratory sample cultures. Supplemental oxygen was required by 52.08% (*n*=25) of the patients, 29.16% (*n*=14) needed NIMV and 22.91% (*n*=11) required IMV. ICU admission was reported at 37.5% (*n*=18), acute kidney injury was reported in 10.41% (*n*=5) of the patients, with a mean hospital stay of 8 days (IQR 13) and in-hospital mortality was 6.5% (*n*=3) (Table 3).

**Fig. 3.**
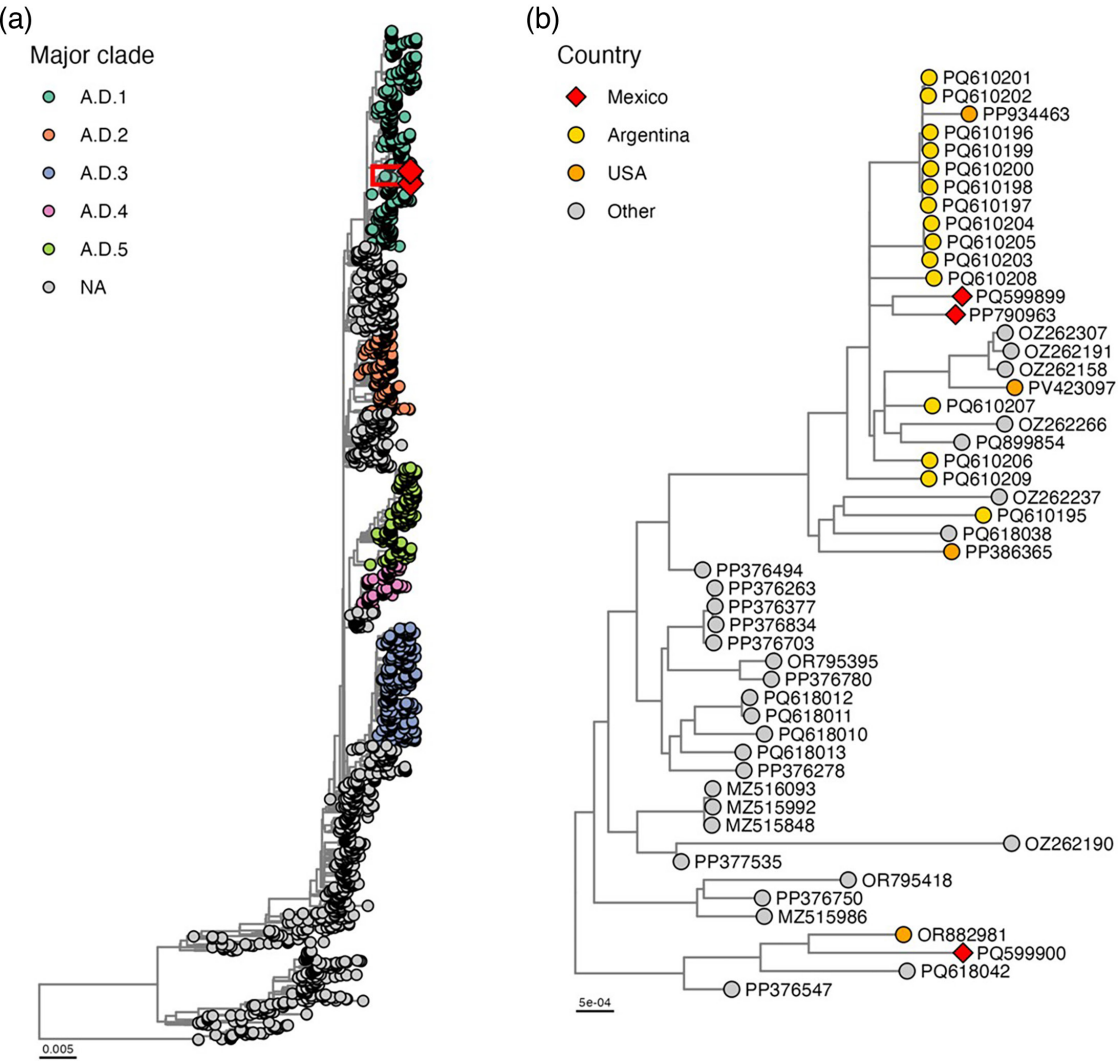
Phylogenetic relationship of RSV. (**a**) Phylogenetic tree with 2,898 whole-genome sequences of RSVA made with Nextstrain’s RSV bioinformatic pipeline. The red box indicates magnification. (**b**) Clade indicating the Mexican isolates and related sequences. The red diamond corresponds to the Mexican sequences from this study. The tree was annotated in ggtree.

We obtained three whole-genome sequences of RSV-A, with a mean depth of 1,290× and 99.8% coverage. The phylogenetic analyses revealed that the obtained sequences clustered in two different clades: two sequences in the clade A.D.1.5 and one in the clade A.D.1 (Fig. 3). The two sequences from Mexico make a monophyletic clade, suggesting a regional distribution. The viruses belonging to the A.D.1.5 clade are distributed throughout America and Europe (Ireland and France), Argentina being the country where the most A.D.1.5 viruses were isolated. The phylogeographic analysis revealed that there was a viral exchange between the USA and Argentina. Afterwards, the virus spread to Mexico and Ireland. The most recent common ancestor of the Mexican phylogenetic clade was estimated to be from Mexico in August 2022. Therefore, there was a previous viral introduction which gave rise to the Mexican clade. On the other hand, the sample from clade A.D.1 makes a monophyletic clade with a sequence from a virus isolated in the USA. The most recent common ancestor of this clade was predicted to be from the USA and was circulating during November 2020.

## Discussion

The present study describes the clinical and genomic surveillance of RSV infection from the northern Mexican region. In our adult patients, the most frequent comorbidities were hypertension and type 2 diabetes. An 8% risk of hospitalization has also been described for patients with chronic obstructive pulmonary disease (COPD) [20], which is aligned with our population, where all three hospitalized patients had a history of COPD. It is previously known that viral infections such as RSV directly affect the respiratory epithelium, such as destruction of ciliated cells, disruption of mucociliary functions and airway inflammation [21,22]. These changes result in airway obstruction and hyperinflation, leading to an accelerated decline in lung function [23]

In the studied population, one in two adult patients required hospitalization, with one-third of them being over 65 years old, which corresponds to the findings of Brosh-Nissimov *et al*. [24], who reported a significant increase in incidence in the population after 65 years (63.9/100,000) and even higher after 75 years (199/100,000).

Adult patients required IMV in 15% of the cases, which aligns with the previous findings of Havers *et al.* [25], who reported 18.5% of 1,634 RSV-hospitalized patients in the USA. Moreover, we observed a higher in-hospital mortality rate than other studies, between 4.7% and 7.2% [26]. However, this increase is associated with preexisting pulmonary functional impairment, a weakened immune response and greater susceptibility to severe complications such as pneumonia, acute exacerbations and respiratory failure. Several epidemiological studies have documented that hospitalization rates associated with RSV infection are higher in infants aged 0 to 2 months and decrease with age [27]. Recent studies describe hospitalization rates of up to 40% [26] in paediatric patients diagnosed with RSV, which is much lower compared to our population. This may be due to selection bias because, in our paediatric patients, screening for viral respiratory infections was carried out in the emergency department, where many had at least 1 day of hospitalization while awaiting results. In our case, most of the hospitalized patients were under 2 years of age, which aligns with findings described by Mori *et al*. [28], where in a cohort of 946 (26.6%) hospitalized with RSV infection, an association with the need for hospitalization was found in children under 2 years [odds ratio 2.46 (1.65–3.67), *P*<0.001].

A systematic review of 42 studies from the USA (1997–2018) showed that among all U.S. infants and children under 5 years with RSV, the mortality rates ranged from 0.04% to 0.55% [29]. Given our smaller sample, this cannot be directly compared with our population.

Furthermore, the RSV serotype in all sequenced samples was RSV-A, consistent with previous reports from Mexico. There is only one genotyping study to date [11,11], which determined a predominance of RSV-A and genotype ON1 in samples from paediatric patients. Previously, the RSV whole genome from Mexican samples had been made. However, these samples were processed in foreign laboratories [30]. These are the first whole-genome sequences of RSV-A from Mexico, after the pandemic. Also, these are the first whole-genome sequences produced from a Mexican laboratory.

Although we initially planned to sequence 54 RSV-positive samples, we were only able to recover 3 complete genomes. Many of the samples had been stored for a long time or had gone through several freeze–thaw cycles, which led to RNA degradation and poor-quality input material. As a result, we could only generate partial sequences from most samples, while only three yielded complete, high-quality genomes suitable for downstream analysis.

Our phylogeographic reconstruction, based on time-scaled trees and the inferred geographic states of ancestral nodes, suggested potential viral exchanges among countries. By integrating collection dates and sampling locations, the analysis estimated the most likely origin of each lineage and the direction of viral spread. Within the A.D.1.5 clade, the two Mexican genomes were placed among viruses from the USA, Argentina and Europe. The ancestral nodes connecting these sequences were inferred to have originated in the USA and Argentina, suggesting that viruses from these regions may have contributed to introductions into Mexico and Europe. The two Mexican genomes formed a distinct monophyletic cluster, compatible with a single introduction around August 2022 followed by local transmission. In contrast, the genome assigned to clade A.D.1 grouped with a U.S. sequence, with its common ancestor inferred to have circulated in the USA around late 2020, compatible with a separate introduction event. Our findings are consistent with the ongoing international circulation of RSV-A lineages, although broader sampling would be required to confirm transmission dynamics.

We acknowledge some limitations in our analysis, particularly the small sample size and the restriction to patients from a single winter season, which may limit the generalizability of our findings.

## Abbreviations

COPD: chronic obstructive pulmonary disease
ICU: intensive care unit
IMV: invasive mechanical ventilation
IQR: interquartile range
NIMV: non-invasive mechanical ventilation
RSV: respiratory syncytial virus
RT-qPCR: reverse transcription quantitative polymerase chain reaction
SARS-CoV-2: Severe Acute Respiratory Syndrome Coronavirus 2
SISPA: sequence-independent single-primer amplification
